# Mesenchymal stem cell derived extracellular vesicles: promising immunomodulators against autoimmune, autoinflammatory disorders and SARS-CoV-2 infection

**DOI:** 10.3906/biy-2002-79

**Published:** 2020-06-21

**Authors:** Özlem BULUT, İhsan GÜRSEL

**Affiliations:** 1 Therapeutic Oligodeoxynucleotide Research Laboratory (THORLAB), Department of Molecular Biology and Genetics, Faculty of Science, İhsan Doğramacı Bilkent University, Ankara Turkey

**Keywords:** Mesenchymal stem cells, extracellular vesicles, exosomes, inflammation, autoimmunity, COVID-19

## Abstract

Discovery of novel and broad-acting immunomodulators is of critical importance for the prevention and treatment of disorders occurring due to overexuberant immune responseincluding SARS-CoV-2 triggered cytokine storm leading to lung pathology and mortality during the ongoing viral pandemic. Mesenchymal stem/stromal cells (MSCs), highly regarded for their regenerative capacities, also possessesremarkable immunoregulatory functions affecting all types of innate and adaptive immune cells. Owing to that, MSCs have been heavily investigated in clinic for the treatment of autoimmune and inflammatory diseases along with transplant rejection. Extensive research in the last decaderevealed that MSCs carry out most of their functions through paracrine factors which are soluble mediators and extracellular vesicles (EVs). EVs, including exosomes and microvesicles, are an efficient way of intercellular communication due to their unique ability to carry biological messages such as transcription factors, growth factors, cytokines, mRNAs and miRNAs over long distances. EVs originate through direct budding of the cell membrane or the endosomal secretion pathway and they consist of the cytosolic and membrane components of their parent cell. Therefore, they are able to mimic the characteristics of the parent cell, affecting the target cells upon binding or internalization. EVs secreted by MSCs are emerging as a cell-free alternative to MSC-based therapies. MSC EVs are being tested in preclinical and clinical settings where they exhibit exceptional immunosuppressivecapacity. They regulate the migration, proliferation, activation and polarization of various immune cells, promoting a tolerogenic immune response while inhibiting inflammatory response. Being as effective immunomodulators as their parent cells, MSC EVs are also preferable over MSC-based therapies due to their lower risk of immunogenicity, tumorigenicity and overall superior safety. In this review, we present the outcomes of preclinical and clinical studies utilizing MSC EVs as therapeutic agents for the treatment of a wide variety of immunological disorders.

## 1. Introduction

### 1.1 Mesenchymal stem/stromal cells (MSCs)

Mesenchymal stem cells, also known as mesenchymal stromal cells (MSCs), are multipotent cells with self-renewal capacity and the ability to differentiate into mesenchymal lineages such as osteogenic, chondrogenic and adipogenic (Pittenger et al., 1999). MSCs can also give rise to other cell types including neurons (Arthur et al., 2008) and hepatocytes (Snykers et al., 2009). According to International Society for Cellular Therapy’s minimal criteria, apart from the self-renewal and tri-lineage differentiation capacities, MSCs are characterized byexpressing surface markers CD73, CD90 and CD105 while they lack CD14, CD19, CD34, CD45 and class II major histocompatibility complex (MHC) molecules (Dominici et al., 2006). 

Although MSCs were first identified inthe bone marrow (Friedenstein et al., 1970), since then they have been isolated from various sources including peripheral blood, umbilical cord tissue Wharton’s jelly, umbilical cord blood, dental pulp, adipose tissue, amniotic fluid, endometrium, placenta and menstrual blood (Ding et al., 2011; Hass et al., 2011). MSCs of different sources vary in differentiation capacities, gene expressions and secretomes (El Omar et al., 2014). For instance, adipose tissue-derived MSCs (AD-MSCs) and umbilical cord-derived MSCs (UC-MSCs) from Wharton’s jelly are stronger immunosuppressors compared to bone marrow-derived MSCs (BM-MSCs) (Melief et al., 2013; Li et al., 2014). MSCs have long been an interest for regenerative medicineowing to their exceptional differentiation capacity. In the past two decades, their ability to interact with the immune system and to modulate immune responses has also attracted a great amount of attention.

### 1.2. Extracellular vesicles (EVs)

Extracellular vesicles (EV), main two classes of which are microvesicles and exosomes, are small vesicles forming by direct budding of the plasma membrane or originating from endosomes, respectively (Stahl and Raposo, 2019). Microvesicles, the bigger class of EVs, can range between 100 and 1000 nm in diameter while exosomes are much smaller and usually in the range of 30 and 100 nm in diameter (Minciacchi et al., 2015). EVs partially enclose the cell’s cytosol with a lipid bilayer and may contain cytosolic or transmembrane proteins, amino acids, lipids, mitochondrial or genomic DNAs, mRNAs, miRNAs and long non-coding RNAs of the parent cell (Maas et al., 2017). EVs are a conserved and highly efficient form of intercellular communication utilized by prokaryotic and eukaryotic cells. In mammalians, they can be found in every bodily fluid including blood, urine, saliva, cerebrospinal fluid, synovial fluid, bronchoalveolar fluid, nasal fluid, amniotic fluid, uterine fluid, breast milk, seminal plasma and bile where they carry out various physiological roles (Yanez-Mo et al,, 2015).

EVs are critical for homeostasis and theyalsogreatly impactdisease pathogenesis and immune defense (Yuana et al., 2013). Due to their unique ability to transmit critical biological information over long distances, EVs have lately been attractive targets for therapeutic and diagnostic purposes.Although most existing preclinical and clinical studies investigate EVs as biomarkers for diagnostic and prognostic purposes, the number of studies utilizing EVs as therapeutic agents has been rapidly growing (Wiklander et al., 2019). In this review, the encompassing term “EV” will be used in cases when the distinct EV types exosomes and microvesicles have not been separated and the vesicle population mentioned in the studies include both types of EVs.

## 2. MSCs as immunomodulators

MSCs are promising therapeutic agents for autoimmune and inflammatory diseases as well as transplant rejection, even though they may promote inflammation in certain cases depending on the factors present in the environment. During tissue injury or inflammation, danger-associated molecular patterns (DAMPs) or pathogen-associated molecular patterns (PAMPs) trigger the recruitment and response of MSCs through Toll-like receptors (TLRs) (Bernardo and Fibbe, 2013). Mouse and human MSC types differentially expressvarious TLRs, most prominently TLR3 and TLR4. An MSC classification was proposedto term MSCs with proinflammatory and antiinflammatory behaviors as MSC1 and MSC2, respectively (Waterman et al., 2010). Upon TLR4 stimulation, MSCs shift to MSC1 state and produce higher amounts of cytokine IL-6 and chemokines CCL5 (RANTES), CXCL8 and CXCL10, thereby trigger immune cell recruitment and activation. On the other hand, upon TLR3 stimulation, MSCs are skewed to the MSC2 state and produce immunosuppressive molecules prostaglandin E2 (PGE2) and indoleamine 2,3-dioxygenase (IDO). PGE2 inhibits IL-2 production from T cells and also suppress granulocyte, natural killer (NK) cell, macrophage and dendritic cell (DC) functions (Kalinski, 2012). IDO is important for tryptophan catabolism and it leads to generation of suppressive tryptophan catabolites that have cytotoxic effects on T-cells (Terness et al., 2002).Through such soluble mediators, MSCs modulate both innate and adaptive arms of immunity.

### 2.1. MSCs and innate immunity

MSCs are able to regulate variousfacets of innate immunity. Upon microbial challenge, MSCs secrete CXCL8 and macrophage migration inhibitory factor (MIF) that cause neutrophilaccumulationand consequently microbial clearance (Brandau et al., 2010). On the other hand, they producesuperoxide dismutase 3 (SOD3) that reduces superoxide anion levels, inhibits neutrophil extracellular trap (NET) formation and the release of tissue-damaging proteases in order to restrain excess tissue damage mediated by neutrophils (Jiang et al., 2016). MSCs also secrete chemokines such as CCL2 (MCP-1), CCL3 and CCL12 that promote monocyte and macrophage migration to wound sites which leads to enhanced healing (Chen et al., 2008). 

Moreover, MSCs restrict allergic inflammation by inhibiting IgE secretion, cytokine production and degranulation of mast cells in a PGE2, transforming growth factor β (TGFβ) and prostaglandin-endoperoxide synthase 2 (COX2)-dependent manner (Brown et al., 2011; Kim et al., 2015). MSCs downregulate activating NK receptors such as NKG2D, inhibit IL-2 induced NK cell proliferation, their cytotoxic activity and cytokine production through IDO and PGE2 (Spaggiari et al., 2008). Upon exposure to MSCs, NK cells also gain CD73 expression which leads to production of adenosine that has antiinflammatory effects (Cronstein, 1994; Chatterjee et al., 2014). 

In DCs, MSCs downregulate CD40, CD80, CD86 and HLA-DR expressions, inhibit their differentiation from monocytes andmaturation, limitcytokine producing and T-cell activating capacities (Zhang et al., 2004). Also, MSCs skew mature DCs into an IL-10-producing regulatory phenotype in an IL-10, suppressor of cytokine signaling 3 (SOCS3) and Jagged-2-dependent manner (Zhang et al., 2009; Liu et al., 2012). IL-6 secreted by MSCs was also suggested to upregulate SOCS1 and drive a similar tolerogenic DC phenotype (Deng et al., 2014). SOCS1 upregulation caused impaired TLR4 signaling and inhibited DC maturation. Another study identified PGE2 as the key mediator produced by MSCs that inhibits DC maturation (Spaggiari et al., 2009). 

Alternatively activated M2 macrophages are another regulatory cell type promoted by MSCs. Macrophages upregulate CD206 and arginase 1 expression, increase IL-4 and IL-10 secretion, reducemonocyte chemoattractant protein 1 (MCP-1), tumor necrosis factor α (TNFα), IL-1β and inducible nitric oxide synthase (iNOS) production when cocultured with MSCs (Zhang et al., 2010; Cho et al., 2014). Mechanisms that have been shown to contribute to MSC-based induction of antiinflammatory M2 phenotype include TSG-6-mediated NF-κB suppression (Choi et al., 2011), PGE2 engagement with EP2 and EP4 receptors on macrophages (Nemeth et al., 2009) and lactate-mediated metabolic reprogramming of macrophages (Selleri et al., 2016). IL-1Ra, another antiinflammatory cytokine secreted by MSCs, was also identified to play a role in M1 to M2 polarization (Luz-Crawford et al., 2016).

### 2.2. MSCs and adaptive immunity

Suppressive actions of MSCs also involve the adaptive arm of the immune system. MSCs cause cell cycle arrest and thus block CD4+ and CD8+ T-cell proliferation (Di Nicola et al., 2002; Glennie et al., 2015), induce T-cell apoptosis via FasL-Fas engagement (Akiyama et al., 2012) and IDO-mediated tryptophan catabolism (Plumas et al., 2005), promote regulatory T cell (Treg) generation and IL-10 production while preventing Th1 and Th17 differentiation of CD4+ T-cells (Luz-Crawford et al., 2013). However, T-cell suppressing abilities of MSCs require presence of inflammatory cytokines such as IFNγ, TNFα, IL-1α or IL-1β in the vicinity. These cytokines trigger the production of T-cell attracting chemokines and iNOSfrom MSCs so that T-cells migrate towards the MSCs and get inactivated by nitric oxide (NO) produced by iNOS (Ren et al., 2008). IDO production from MSCs also increase upon exposure to IFNγ, IL-6 and TNFα (Crop et al., 2010). Of note, IDO is the key mediator of MSC-mediated immunosuppression in humans and other primates, while iNOS takes on the same rolefor rodent MSCs (Su et al., 2014).

Similar to their effects on T-cells, MSCs also block B-cell proliferation via cell cycle arrest, inhibit their antibody production, and hinder their chemotactic abilities by downregulating chemokine receptors that they expresssuch as CCR7, CXCR4 and CXCR5 (Corcione et al., 2006). MSCs also compromise B-cell viability by regulating ERK1/2 and p38 pathways (Tabera et al., 2008). IL-1Ra, which contributes to MSC-induced M2 macrophage polarization, is also important for preventing B-cell differentiation into plasma cells (Luz-Crawford et al., 2016). Another mechanism through which MSCs suppress B-cell function is the engagement of programmed death 1 (PD-1) receptor on B cells with PD-1 ligand (PD-L1)on MSCsupon direct contact between the cells and theresulting suppression is more potent in the presence of IFNγ (Schena et al., 2010). Lastly, MSCs are also able to promote the generation of IL-10 producing regulatory B-cells (Bregs) (Franquesa et al., 2015).

### 2.3. Clinical use of MSCs 

MSCs have been heavily investigated in clinical trials for many conditions. Currently there are over 900 completed, ongoing and planned trials related to MSCs (Clinicaltrials.gov, 2019)^1^1U.S. National Library of Medicine (2019). ClinicalTrials.gov [online]. Website https://clinicaltrials.gov/ct2/results?cond=&term=mesenchymal+stem+cells&cntry=&state=&city=&dist= [accessed 31 August 2019].. The wide range of these trials involve cardiovascular, rheumatological, neurological, hematological, autoimmune and inflammatory disorders along with transplant rejection. Due to their far-reaching immunomodulatory capacities, MSCs have shown great promise for the treatment of immunological diseases.

BM-MSC treatment could regress graft-versus-host disease (GvHD), a severe transplant rejection that occurs after hematopoietic stem cell (HSC) transplants, irrespective of MHC compatibility of the receiver and the MSC donor (Le Blanc et al., 2008). MSC-administered GvHD patients had higher Treg numbers without an increased risk of tumor relapse or viral infections (Zhao et al., 2015). When patients undergoing kidney transplants were inoculated with autologous BM-MSCs, incidence of acute rejection and opportunistic infections were lower while renal function recovery was faster (Tan et al., 2012). Calcineurin inhibitors are used as immunomodulating agents for transplantation but they have nephrotoxic effects. Addition of BM-MSC treatment allowed the use of lower doses of these nephrotoxic substances upon renal transplantation with no side effects (Pan et al., 2016). When cotransplanted with pancreatic islet cells to type 1 diabetes patients, MSCs enhanced graft vascularization while reducing autoimmune reaction through T cell and DC inhibition (Figliuzzi et al., 2014).

MSCs also showed remarkable immunosuppressive potential in inflammatory and autoimmune disorders. Intrafistular autologous BM-MSC injection to fistulizing Crohn’s disease patients promoted mucosal healing and reduced disease activity which were attributable to a local and systemic increase in Treg numbers due to MSC treatment (Ciccocioppo et al., 2011). Intravenous (IV) injection of allogeneic BM-MSCs to treatment-refractory patients with luminal Crohn’s disease also led to significant clinical improvement without causing any adverse effects (Forbes et al., 2014). A single subcutaneous injection of UC-MSCs could decrease circulating eosinophil numbers and IgE levels and lead to clinical improvement in atopic dermatitis patients (Kim et al., 2017). Moreover, allogeneic BM-MSC infusion for drug-resistant severe systemic lupus erythematosus (SLE) decreased the rates of organ dysfunction and disease relapse (Wang et al., 2013). In amyotrophic lateral sclerosis (ALS) and multiple sclerosis (MS) patients, intrathecal or IV BM-MSC injection increased Treg numbers, reduced lymphocyte proliferation and downregulated the expression of activation and maturation markers such as CD40, CD83 and HLA-DR on DCs (Karussis et al., 2010). 

Contribution of MSCs in infectious diseases is also being investigated. On one hand, literature findings revealed that these cells might play a critical role in spreading the disease during onset of bacterial invasion. On the other hand, data indicates that pathogen driven immune exacerbation could be alleviated by these cells and could act as an effective tool for treatment of infectious diseases (Marrazzo et al., 2019). MSCs were shown to be effective against endotoxin induced sepsis, or acute lung injury or even microbe triggered pneumonia (Marrazzo et al., 2019). In the case of the current coronavirus disease 2019 (COVID-19) pandemic triggered by SARS-CoV-2 infection, however, the infection leads to lung pathology (Liue et al., 2020) and utilization of MSCs to control the clinical outcomes were shown to be effective on patients with COVID-19 pneumonia (Leng et al., 2020). In a pilot study conducted in China, TNFα levels were significantly decreased while regulatory DCs and IL-10 levels were elevated in MSC-treated patients. SARS-CoV-2 enters to target cells via surface receptor ACE2 and also in some cells requiresTMPRSS2 (Hoffmann et al., 2020). Since MSCs are negative for both ACE2 and TMPRSS2, they would be free from COVID-19 infection, thus could be safe and beneficial for the patients with COVID-19 (Zhao, 2020). As of April 9, 2020, out of 583 registered clinical trials, 24 are being conducted with MSCs against COVID-19 (Chinese Clinical Trial Registry, 2020)^2^2Chinese Clinical Trial Register (2020). Chinese Clinical Trial Registry (ChiCTR) [online]. Website u1dbb [accessed 9 April 2020]..

## 3. MSC EVs as the cell-free alternative to MSC-based therapeutics

**Figure 1:**
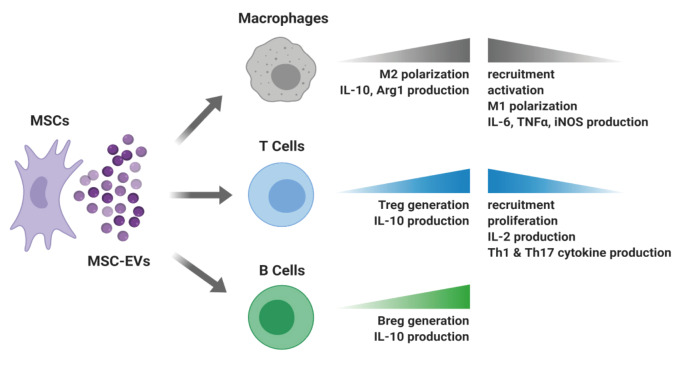
Main mechanisms through which MSC EVs shape immune cell functions to alleviate inflammation in vivo.

The remarkable capacity of MSCs for tissue repair and regeneration has long been attributed to their differentiation ability to other cell types upon environmental cues. However, later it became clear that MSCs exert most of their reparative, angiogenic and immunosuppressive functions through paracrine mediators involving soluble factors and EVs (Caplan and Dennis, 2006). Therefore, MSC-derived EVs, particularly exosomes, are now of great interest as a cell-free alternative to MSC-based therapeutic approaches. Since EVs are able to carry biological messengers such as growth factors, cytokines and miRNAs produced by the parent cell which could shape the behavior of immune cells in the environment (Figure 1), MSC EVs are expected to exert clinically valuable immunomodulatory and regenerative actions similar to their parent cells. 

**Figure 2:**
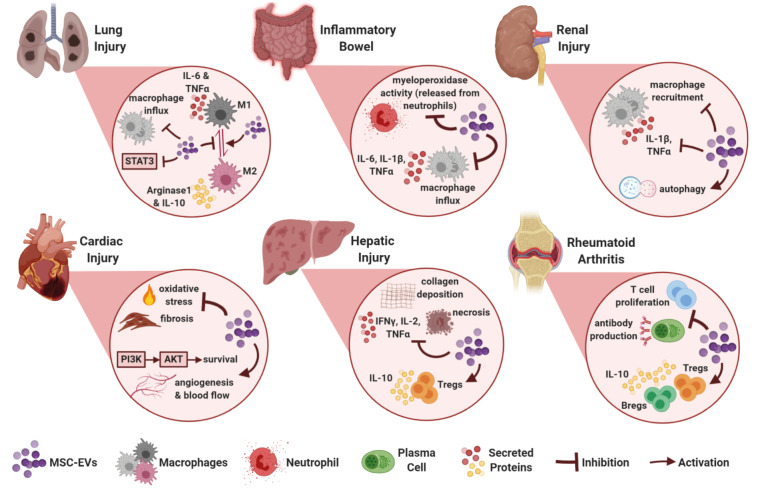
Tissue-specific targets and effects of MSC EVs identified in preclinical disease models.

Therapeutic efficacy of MSC EVs was first reported in a murine myocardial ischemia/reperfusion (I/R) injury model where exosomes from human embryonic stem cell (ESC)-derived MSCs were cardioprotective and able to reduce the infarct size (Lai et al., 2010). Since then, various animal disease models proved that MSC EVs have regenerative and immunosuppressive effects (Figure 2) and use of MSC EVs progressed to clinical trials for the treatments of acute ischemic stroke, refractory macular holes, type 1 diabetes mellitus and bronchopulmonary dysplasia (Clinicaltrials.gov, 2019)^3^3U.S. National Library of Medicine (2019). ClinicalTrials.gov [online]. Website https://clinicaltrials.gov/ct2/results?cond=&term=mesenchymal+stem+cell+exosome&cntry=&state=&city=&dist= [accessed 30 August 2019]..

### 3.1. MSC EVs in cardiac injury

Following the abovementioned first I/R injury study, in another murine model, exosomes secreted by human ESC-derived MSCs reduced oxidative stress, increased myocardial viability through the activation of PI3K/Akt pathway, and consequently enhanced cardiac function (Arslan et al., 2013). BM-MSC EVs or exosomes also promoted angiogenesis and provided enhanced blood flow in myocardial infarction models while inhibiting T-cell proliferation (Bian et al., 2014; Teng et al., 2015). In an interesting study, rats with myocardial infarction were treated with cardiac stem cells (CSCs) that were previously exposed to BM-MSC exosomes in culture. CSCs exposed to MSC exosomes led to greater improvement of cardiac function and reduction of fibrosis compared to regular CSC treatment (Zhang et al., 2016). 

### 3.2. MSC EVs in renal injury

MSC-EVs also proved to be beneficial for the treatment of renal injuries. BM-MSC exosomes were protective in a murine renal I/R injury model and the protective effect was dependent on the chemokine receptor CCR2 carried on exosome surface which could sequester the chemokine CCL2 and therefore impair macrophage recruitment and activation (Shen et al., 2016). In a study of cisplatin-induced nephrotoxicity, UC-MSC exosomes reduced the levels of proinflammatory cytokines IL-1β and TNFα while promoting autophagy (Wang et al., 2017). 

### 3.3. MSC EVs in hepatic injury

MSC-EVs were reported to alleviate hepatic inflammation and fibrosis. Exosomes from human UC-MSCs were shown to reduce hepatic inflammation while decreasing TGFβ levels and collagen deposition in a murine liver fibrosis model (Li et al., 2013). In mice with concanavalin A (ConA)-induced liver injury, repeated BM-MSC exosome injection reduced IFNγ, IL-1, IL-2, TNFα expression, increased Treg numbers and alleviated necrosis in the liver (Tamura et al., 2016). 

### 3.4. MSC EVs in lung injury

Immunomodulatory properties of MSC-EVs are also therapeutic for lung injury. In mice withhypoxic pulmonary hypertension, BM-MSC exosomes decreased macrophage influx, suppressed inflammatory mediators such as MCP-1 and inhibited STAT3 signaling (Lee et al., 2012). UC-MSC exosomes improved lung structure and function in mice with bronchopulmonary dysplasia by promoting an M1-M2 shift in macrophages evidenced by reduced IL-6, TNFα, CCL5 and increased arginase 1 (Willis et al., 2018). Inhibition of the inflammatory M1 macrophage phenotype and promotion of M2 phenotype was also proposed as the mechanism by which AD-MSC exosomes alleviated inflammation of white adipose tissue in obese mice. Exosome treatment to these mice led to higher arginase 1 and IL-10 expression while limiting IL-6, IL-12 and TNFα (Zhao et al., 2018). Furthermore, although there are no studies using MSC EVs in a preclinical or clinical model of COVID-19, given the promise of intravenous MSC injection for alleviating SARS-CoV-2 infection, it would be worthwhile to investigate the potential of MSC EVs to repair lung injury in COVID-19 patients.

### 3.5. MSC EVs in autoinflammatory disorders

MSC-EVs have been showing promise for the treatment of various autoimmune disorders. AD-MSC exosomes were utilized in a murine type-1 autoimmune diabetes mellitus model where they were able to reduce IFNγ and IL-17 levels while upregulating IL-4, IL-10 and TGFβ along with higher number of Treg cells in spleen (Nojehdehi et al., 2018). In another study that combined type-1 diabetes and experimental autoimmune uveoretinitis (EAU) models, MSC EVs prevented disease onset and inhibited the activation of antigen-presenting cells as well as secretion of Th1 and Th17 cytokines (Shigemoto-Kuroda et al., 2017). Another supporting EAU study revealed that UC-MSC exosomes reduce Th1 and Th17 in the eye, prevented macrophage and T cell infiltration, thereby mitigating disease intensity (Bai et al., 2017).

### 3.6. MSC EVs in inflammatory bowel disease

MSC EVs are also attractive therapeutic agents for inflammatory bowel disease. BM-MSC exosomes reduced tissue damage, limited myeloperoxidase activity in colon, decreased IL-1β, TNFα and iNOS levels in the tissue in a rat model of 2,4,6-trinitrobenzene sulphonic acid (TNBS)-induced colitis (Yang et al., 2015). Likewise, in a mouse model of dextran sulfate sodium (DSS)-induced colitis, upon UC-MSC exosome treatment, macrophage infiltration to the tissue was reduced along with downregulation of IL-1β, IL-6, IL-7, TNFα and iNOS in colon tissue and spleen (Mao et al., 2017).

### 3.7. MSC EVs in other inflammatory conditions

UC-MSC EVs were able to reduce mortality in mice that received allogeneic HSC transplantation. Treated mice had fewer T cells and lower serum levels of IFNγ, IL-2, TNFα and higher levels of IL-10 (Wang et al., 2016). In mice with spinal cord injury, UC-MSC exosomes led to lower levels of IFNγ, IL-6, TNFα, macrophage inflammatory protein-1 (MIP-1α) and improved the recovery of animals (Sun et al., 2018). More recently, potential of MSC exosomes for alleviating rheumatic diseases was also revealed. In a collagen-induced arthritis (CIA) study, BM-MSC exosomes inhibited antibody production and T-cell proliferation in vitro while enhancing Breg and Treg populations in vivo where they were able to alleviate disease progression (Cosenza et al., 2018). 

### 3.8. MSC EV therapeutics in humans

Despite numerous publications of preclinical studies depicting MSC EVs as safe and remarkably effective therapeutic agents for a wide-range of disorders, there are only two reports of clinical success utilizing MSC EVs thus far. The first one was a one-patient trial where BM-MSC exosomes were administered to a therapy-refractory GvHD patient (Kordelas et al., 2014). IFNγ, IL-1β and TNFα produced by the patient’s peripheral blood mononuclear cells (PBMCs) were reduced more than 50% after the third exosome injection. Clinical symptoms of the patient improved exceptionally and were stable for 4 months although the patient later died of pneumonia. In the other study where 20 chronic kidney disease patients received UC-MSC EVs, improved clinical conditions were accompanied with reduced TNFα and elevated IL-10 and TGFβ levels in circulation (Nassar et al., 2016).

## 4. Conclusions

Outcomes of numerous preclinical and clinical studies presented in this review leave no doubt of the efficacy of MSC EVs as immunosuppressive agents similar to their parent cells. Although EV-based therapies are certainly attractive in terms of safety, there are a few concerns that should be addressed for proper clinical use. In the MSC EV studies, there is wide variation and lack of standardization regarding MSC expansion and exosome or EV purification methods. Moreover, as Kordelas et al. (2014) pointed out, EVs of MSCs from different donors might differ in terms of cargo and therefore immunomodulatory capacity. Thus, potency tests and protocol standardization are critical for mass-production of clinical-grade exosome or EV preparations.

MSC EVs appear to be beneficial for the treatment of GvHD, cardiovascular, renal, hepatic and pulmonary diseases, autoimmune diseases such as type 1 diabetes and uveitis, inflammatory bowel diseases and rheumatoid arthritis (Figure 2) through mechanisms including inhibition of T cell proliferation, Th1 and Th17 differentiation, macrophage recruitment, M1 polarization, as well as promotion of Treg cells, Breg cells and M2 polarization (Figure 1). However, further investigation is essential in order to fully reveal the mechanisms of immunomodulation and the EV cargo responsible for these cellular alterations specific for each clinical condition.Profiling of EV cargo with approaches such as proteomics, lipidomics and RNA-sequencing is crucial to fill the knowledge gap. Future in vitro and preclinical research and newly commencing clinical trials will surely establish MSC EVs as a novel, safe and effective therapeutic approach for the treatment of immunological disorders. Lastly, in the course of the devastating COVID-19 pandemic, use of MSC derived EVs in the treatment of COVID-19 driven pneumonia and lung pathology should be investigated as a viable and safe strategy since MSCs are not subject to SARS-CoV-2 infection and corresponding EVs will be devoid of any virus or virus by-products.

**Acknowledgments**

Özlem Bulut was funded by TÜBİTAK project number 115S837. Figures were created with Biorender.com.
